# UGT76B1 and 41 Additional Arabidopsis UDP-Glycosyltransferases Show No Detectable In Vitro Glycosylation Activity Toward *N*-Hydroxypipecolic Acid

**DOI:** 10.3390/life16060992

**Published:** 2026-06-12

**Authors:** Jiyuan Bao, Taiga Uchiyama, Kazuki Kusunoki, Yuka Shinohara, Yurika Tanigawa, Megumi Watanabe, Nanami Sakata, Hidenori Matsui, Kazuhiro Toyoda, Yuki Ichinose, Yoshiteru Noutoshi

**Affiliations:** 1 Graduate School of Environmental, Life, Natural Science and Technology, Okayama University, Okayama 700-8530, Japan; jybao629@gmail.com (J.B.); nanami.sakata@okayama-u.ac.jp (N.S.); hmatsui@okayama-u.ac.jp (H.M.); pisatin@okayama-u.ac.jp (K.T.); yuki@okayama-u.ac.jp (Y.I.); 2 School of Agriculture, Okayama University, Okayama 700-8530, Japan

**Keywords:** UGT76B1, *N*-hydroxypipecolic acid (NHP), salicylic acid (SA), glycosyltransferase, plant immunity

## Abstract

*N*-hydroxypipecolic acid (NHP) is a key mobile signal in systemic acquired resistance in plants, and its glycosylation has been proposed to regulate immune signaling. Previous studies have demonstrated that the UDP-glycosyltransferase UGT76B1, known as an SA glycosyltransferase in *Arabidopsis thaliana*, also catalyzes NHP glycosylation. In this study, we re-evaluated NHP glycosylation activity of UGT76B1 using an in vitro enzyme-coupled fluorescence assay that quantitatively detects UDP released during UDP-sugar-dependent glycosylation. Unexpectedly, our biochemical analyses demonstrated that UGT76B1 lacks genuine glycosylation activity toward NHP under the in vitro assay conditions tested, although this system clearly detected UGT76B1 activity toward salicylic acid (SA), as well as the activities of UGT74F1 and UGT72B1 toward SA and hydroquinone, respectively. To explore potential UGTs responsible for NHP glycosylation, we evaluated the enzymatic activities of 41 UGT candidates successfully expressed in *Escherichia coli*, which are selected based on transcriptomic responses to tenoxicam treatment, molecular docking simulations using AlphaFold3/AutoDock Vina, phylogenetic criteria, and previous reports. Within this selected and successfully expressed UGT panel, none exhibited authentic NHP glycosylation activity, although this does not preclude the possibility that other members of the Arabidopsis UGT family possess NHP glycosyltransferase activity. Our findings challenge the prevailing view that UGT76B1 is the primary glycosyltransferase for NHP in *A. thaliana* and indicate that NHP metabolism may rely on undiscovered non-canonical enzymes or distinct metabolic pathways that warrant further investigation.

## 1. Introduction

Plants have evolved a sophisticated immune system to defend against potential pathogens. Defense responses are initiated when pattern recognition receptors perceive pathogen- or microbe-associated molecular patterns (PAMPs/MAMPs) and activate pattern-triggered immunity (PTI) [1]. Pathogens can suppress PTI by releasing small secreted proteins, so-called effectors, thereby achieving proliferation in host tissues. In contrast, host plants have developed sensing mechanisms for pathogen effectors or their activities to induce effector-triggered immunity (ETI), a strengthened and prolonged version of PTI associated with the hypersensitive response at the infection site that can overcome pathogen invasion [2]. This immune activation primes distal tissues for enhanced defense, a phenomenon termed systemic acquired resistance (SAR). Several small molecules are involved in coordinating this process, particularly the ubiquitous hormone salicylic acid (SA) [3] and the recently discovered mobile signaling molecule *N*-hydroxypipecolic acid (NHP) [4,5,6,7].

NHP is a naturally occurring non-proteinogenic amino acid-derived metabolite detected in multiple plant families. Previous studies have shown that NHP induces SAR in diverse species, including Arabidopsis, tobacco, sweet pepper, tomato, soybean, corn, and Chinese cabbage [5,6,7,8]. The biosynthetic pathway of NHP in *A. thaliana* is well-characterized: briefly, the aminotransferase AGD2-like defense response protein 1 (ALD1) converts L-Lys to 2,3-dehydropipecolic acid (2,3-DP), which is subsequently reduced to pipecolic acid (Pip) by SAR-deficient 4 (SARD4) [9,10]. Pip is then hydroxylated to NHP by flavin-dependent-monooxygenase 1 (FMO1) [5,6]. Notably, the expressions of *ALD1*, *SARD4*, and *FMO1* are upregulated by pathogen infection, leading to the rapid accumulation of NHP [11]. Furthermore, NHP regulates the transcription of its own biosynthetic genes via a positive feedback loop [5]. The biosynthesis of NHP and the isochorismate synthase (ICS) pathway for SA are both pathogen-inducible and regulated by shared transcriptional regulators, such as *ENHANCED DISEASE SUSCEPTIBILITY 1 (EDS1)*/*PHYTOALEXIN DEFICIENT4* (*PAD4*) and *SAR-deficient 1* (*SARD1*)/*CaM*-*binding protein 60 g* (*CBP60g*) in *A. thaliana* [12,13]. Moreover, NHP-induced transcription requires the SA signaling pathway, including the *NONEXPRESSOR OF PATHOGENESIS-RELATED GENES 1* (*NPR1*) receptor and *TGACG*-*motif*-*binding* (*TGA*) transcription factors [14,15]. Collectively, these findings indicate that the establishment of SAR relies on the synergistic action of SA and NHP.

Chemical modifications, such as methylation, hydroxylation, glycosylation, amino acid conjugation, sulfonation, and carboxylation, are critical dynamic mechanisms for modulating signaling molecules in plants [16]. Methyl salicylate (MeSA) functions as a volatile signal that activates disease resistance in neighboring plants [17,18], while SA 3-hydroxylase (S3H) is involved in SA catabolism and regulates leaf longevity in *A. thaliana* [19]. Conversely, the glycosylation of SA results in the formation of SA-*O*-β-D-glucoside (SAG) and SA glucose ester (SGE), which are generally considered inactive forms functioning in storage or further metabolism in plants [20]. Notably, both NHP and NHP-*O*-β-D-glycoside (NHPG) have been detected following pathogen inoculation or exogenous application of NHP in *A. thaliana*, suggesting that NHPG may serve as an inactive form for storage or catabolism in plants [5].

To date, 123 uridine diphosphate (UDP)-glycosyltransferase (UGT) genes have been identified in the *A. thaliana* genome, according to the UGT Nomenclature Committee (https://labs.wsu.edu/ugt (accessed on 21 May 2026)). However, most of these UGTs have not been fully characterized with respect to their biochemical activities and biological functions [21,22]. Among those that have been characterized, *UGT74F1* and *UGT74F2* contribute to resistance against *Pseudomonas syringae* pv. *tomato* DC3000 (*Pst*) by regulating free SA content through glycosylation [20,23,24]. Similarly, *ugt73b3* and *ugt73b5* mutants display decreased resistance to *Pst*, although their specific substrates remain unclear [25]. Additionally, UGT72B1 catalyzes the glucose conjugation of monolignols, which is essential for normal cell wall lignification [26]. Most notably, UGT76B1 has been identified as a promiscuous glycosyltransferase that recognizes multiple substrates, including SA, isoleucic acid (2-hydroxy-3-methyl-pentanoic acid, ILA), and NHP, suggesting that it acts as a central regulator in SAR [27,28,29,30,31,32].

In this study, we re-evaluated the previously reported activities of UGT76B1 using a validated, highly sensitive enzyme-coupled fluorescence assay system. This assay system successfully detected the glycosylation activities of UGT76B1 and UGT74F1 toward SA, as well as that of UGT72B1 toward hydroquinone. However, the glycosylation activity of UGT76B1 toward NHP was unexpectedly below the limit of detection. To identify potential NHP glycosyltransferases, we further evaluated 41 UGTs selected from 68 candidates based on transcriptomic responses to tenoxicam (TNX) [33] and molecular docking simulations, and assessed their in vitro enzymatic activities. However, no catalytic activity for NHP glycosylation was detected in the tested panel. Collectively, our results suggest that UGT76B1 is unlikely to be the primary glycosyltransferase for NHP in *A. thaliana*, or that its activity requires in vivo modifications or cofactors.

## 2. Materials and Methods

### 2.1. Cloning, Expression, and Purification of Recombinant UGT Proteins

Codon-optimized synthetic genes of candidate UGTs were synthesized and cloned into the pET-28a(+) vector acquired from Twist Bioscience (South San Francisco, CA, USA). The constructs were transformed into *E. coli* ArcticExpress (DE3; Agilent Technologies, Santa Clara, CA, USA) competent cells following the manufacturer’s instructions. The codon-optimized nucleotide sequences translate into amino acid sequences that are identical to the corresponding wild-type Arabidopsis proteins. The nucleotide sequences used for heterologous protein expression in this study are provided in Table S1. The recombinant UGT proteins contained an N-terminal 6 × His-tag.

For protein expression, a small amount of recombinant *E. coli* strain culture stored at −80 °C was inoculated into 5 mL of LB liquid medium containing 20 mg/L kanamycin and cultured at 37 °C, 180 rpm for 16 h. The following day, 200 μL of this culture was inoculated into 200 mL of fresh LB liquid medium and cultured at 25 °C, 120 rpm for 3 h. After induction with 100 μM isopropyl β-D-1-thiogalactopyranoside at 10 °C for 24 h, bacterial cells were harvested by centrifugation at 4000 rpm for 10 min at 4 °C and stored at −80 °C until further processing. The purity and integrity of all prepared recombinant UGT proteins were verified by SDS-PAGE (Figure S6).

To isolate soluble UGT proteins, cell pellets were resuspended with xTractor Buffer (Takara Bio, Kusatsu, Japan) and purified using the HisTALON Gravity Column Purification Kit (Takara Bio, Kusatsu, Japan), following the manufacturer’s instructions. To prevent the oxidation of free sulfhydryl residues while avoiding the interference of dithiothreitol (DTT) with cobalt resin binding, 10 mM 3-mercapto-1,2-propanediol was added to the equilibration, wash, and elution buffers as a reducing agent. Fractions containing the target protein were collected and subsequently desalted using Zeba Spin Desalting Column (7K MWCO; Thermo Scientific, Waltham, MA, USA) to remove imidazole. The purified products were concentrated using Amicon Ultra centrifugal filters (Ultracel—30 K; Merck Millipore, Burlington, MA, USA) and exchanged into storage buffer (40 mM Tris-HCl (pH 7.5), 2 mM DTT, and 10% glycerol) to a final volume of approximately 200 μL. Protein purity was assessed by SDS-PAGE using NuPAGE Bis-Tris Mini Protein Gels (4–12%, 1.0 mm; Thermo Scientific, Waltham, MA, USA), and concentrations were quantified by Bradford assay using the Quick Start Bovine Serum Albumin (BSA) Standard Set (Bio-Rad, Hercules, CA, USA).

### 2.2. Glycosyltransferase Activity Assay

Glycosyltransferase activity was determined using an enzyme-coupled fluorescence assay adapted from Kumagai et al. [34] (Figure S1). A total of 150 ng recombinant UGT proteins were incubated in glycosylation reaction buffer (200 μM substrate, 100 μM UDP-glucose (UDP-Glc), 5 mM phosphate buffer (pH 7.3), 2 mM DTT, 10 mM MgCl_2_, and 0.1% (*v*/*v*) Triton X-100) with a total volume of 50 μL. The reaction was performed in 0.2 mL 8-strip PCR tubes using a T100 Thermal Cycler (Bio-Rad, Hercules, CA, USA) at 37 °C for 2 h and terminated by heat inactivation at 95 °C for 5 min. Fluorescence detection was conducted in two steps as follows: first, 50 μL of fluorescence reaction buffer 1 (50 mM Tris-HCl (pH 7.5), 100 μM ATP, 2 U/mL NDP kinase, 0.01% (*w*/*v*) BSA, and 0.02% (*v*/*v*) Triton X-100) was added to the glycosylation reaction mixture in the same tubes and incubated at 25 °C for 1 h; subsequently 100 μL of fluorescence reaction buffer 2 (50 mM Tris-HCl (pH 7.5), 2 mM glucose, 2 U/mL ADP-hexokinase, 2 U/mL glucose-6-phosphate (G6P) dehydrogenase, 2 U/mL diaphorase, 200 μM NADP^+^, 200 μM resazurin, 20 mM N-ethylmaleimide, 0.01% (*w*/*v*) BSA and 0.02% (*v*/*v*) Triton X-100) was added, followed by a second incubation at 25 °C for 1 h. The total volume of the reaction mixture was transferred to a new 0.5 mL PCR tube, and the fluorescence intensity due to the formation of resorufin was subsequently measured using a DS-11 Fluorometer (DeNovix, Wilmington, DE, USA) with excitation at 540 nm and emission at 590 nm.

UDP standard curves (0–100 μM) were generated in the presence of SA, NHP, Pip, 3,4-DHBA, and hydroquinone (Figure S2a–e). Among the tested compounds, only hydroquinone caused concentration-dependent quenching of the fluorescence signal (Figure S2e). To ensure the accuracy of the steady-state kinetic parameters, we developed and applied a mathematical correction method for hydroquinone. UDP standard curves obtained in the presence of defined concentrations of hydroquinone (50, 100, and 200 μM) or mock (H_2_O) were analyzed (Figure S2e). The signal inhibition rate was then determined for each hydroquinone concentration using the following formula: Inhibition rate = 1 − (Slope_hydroquinone_/Slope_mock_). Plotting the derived inhibition rates against the corresponding hydroquinone concentrations revealed a highly linear relationship (R^2^ = 0.9978; Figure S2f). The specific inhibition rate at each substrate concentration was interpolated using this linear regression model, and the raw fluorescence data were then corrected by dividing them by the corresponding (1 − inhibition rate) factor before Michaelis–Menten curve fitting. The limit of detection (LOD) and limit of quantification (LOQ) were determined based on the standard deviation of the blank response and the slope of the UDP standard curve. Specifically, LOD and LOQ were calculated using the formulas LOD = 3.3 × σ/S and LOQ = 10 × σ/S, respectively, where σ represents the standard deviation of the blank (UDP = 0 μM) and S represents the slope of the UDP standard curve. In this study, the LOD and LOQ were 0.091 and 0.28 nmol, respectively. To represent true signal-to-noise ratios, we included parallel no-acceptor substrate controls in all experimental groups. Authentic enzymatic activities were evaluated by statistically comparing the raw RFU values of the experimental groups directly with those of their corresponding no-acceptor-substrate controls.

### 2.3. Plant Materials and Growth Conditions

*A. thaliana* Col-0 (wild-type) seeds were stratified at 4 °C for 72 h and sown on half-strength Murashige–Skoog (MS) medium supplemented with 1% (*w*/*v*) sucrose and 1% (*w*/*v*) agar. The plates were incubated at 23 °C under long-day conditions (16 h light/8 h dark cycle) for 1 week. Germinated seedlings were transferred to soil (Super mix A; Sakata seed corporation, Yokohama, Japan) and grown for 4–5 weeks under the same conditions.

### 2.4. Leaf Infiltration, RNA Extraction and Quantitative RT-PCR

Leaf infiltration was performed as described by Yildiz et al. [15]. Briefly, 1 mM NHP or SA (sodium salicylate) solutions were infiltrated into three rosette leaves of 5- to 6-week-old plants from the abaxial side using a needleless syringe. The pH of both the NHP and SA solutions was approximately 6.0. Mock infiltration with sterile distilled water served as a control. Treated rosette leaves and untreated upper leaves were harvested separately at 8 and 24 h post treatment (hpt). For each sample, 10 leaf disks were excised using a 4 mm biopsy punch (~50 mg) and immediately frozen in liquid nitrogen and stored at −80 °C until further processing.

Total RNA samples were extracted using the ISOSPIN Plant RNA (NIPPON GENE, Tokyo, Japan) following the manufacturer’s instructions, including a DNase I digestion step. RNA concentration and purity were quantified using a DS-11 spectrophotometer (DeNovix). First-strand cDNA was synthesized from 500 ng of total RNA using the PrimeScript RT reagent Kit with gDNA Eraser (Perfect Real Time; Takara Bio). Quantitative PCR was performed using Taq Pro Universal SYBR qPCR Master Mix (Vazyme, Nanjing, China) on a LightCycler 96 system (Roche, Basel, Switzerland). Primers for quantitative RT-PCR are as follows: for *PR1*, 5′-CGGAGCTACGCAGAACAACT-3′ and 5′-CTCGCTAACCATGTTCA-3′; for *UBQ10*, 5′-AGATCCAGGACAAGGAGGTATTC-3′ and 5′-CGCAGGACCAAGTGAAGAGTAG-3′.

### 2.5. Differential Expression Analysis

RNA sequencing and differential expression analyses in *A. thaliana* following TNX treatment have been described previously [33], and the sequence data are available from the NCBI Gene Expression Omnibus (GEO) under accession number GSE154593. Briefly, 14-day-old *A. thaliana* (Col-0) seedlings were treated with TNX for 1 day, with three biological replicates. Previous transcriptome analyses revealed that TNX acts as an immune inhibitor by regulating SA-inducible, NPR1-dependent gene expression [33]. Given the extensive synergistic interactions between SA and NHP, we hypothesized that TNX-responsive UGTs might serve as promising candidates for NHP glycosylation. In this study, differentially expressed UGTs were selected as candidates based on thresholds of log2 fold change > 1 for upregulation or <−1 for repression, as well as a false discovery rate (FDR) of 0.001.

### 2.6. Molecular Docking Simulation

The 3D structure models of all UGTs in *A. thaliana* were generated using AlphaFold3. To identify putative substrate-binding pockets and key amino acid residues involved in ligand interactions, the predicted UGT structures were structurally aligned with the crystal structure of UGT74F2 in complex with UDP and SA (PDB ID: 5U6M) [20] using PyMOL (v 3.1.3.1) (Schrödinger, LLC, New York, NY, USA). Subsequently, molecular docking of NHP to potential amino acid residues forming the substrate-binding pocket of each UGT was performed using AutoDock Tools (v 1.5.6) and AutoDock Vina (v1.2.7). For docking, NHP was prepared from *N*-hydroxy-L-pipecolic acid (PubChem CID; 92246037) as NHP_2S_carboxylate, representing the 2S stereoisomer with a deprotonated carboxylate group. The *N*-hydroxy group was modeled in its protonated form, while the ring nitrogen was left unprotonated. Hydrogen atoms were retained during ligand preparation, and the structure was converted to PDBQT format for docking. Potential amino acid residues forming the acceptor-substrate binding pockets of the UGTs used for binding-affinity calculations are listed in Table S2. Candidates were selected for further in vitro activity analysis based on two criteria: (1) a calculated binding energy lower than −6.0 kcal/mol; (2) manual confirmation showing that NHP was accommodated within the putative acceptor-substrate binding pocket, in proximity to the histidine residue at the reaction center.

## 3. Results

### 3.1. Validation of the In Vitro Assay System for Measuring Glycosyltransferase Activity

**Figure 1:**
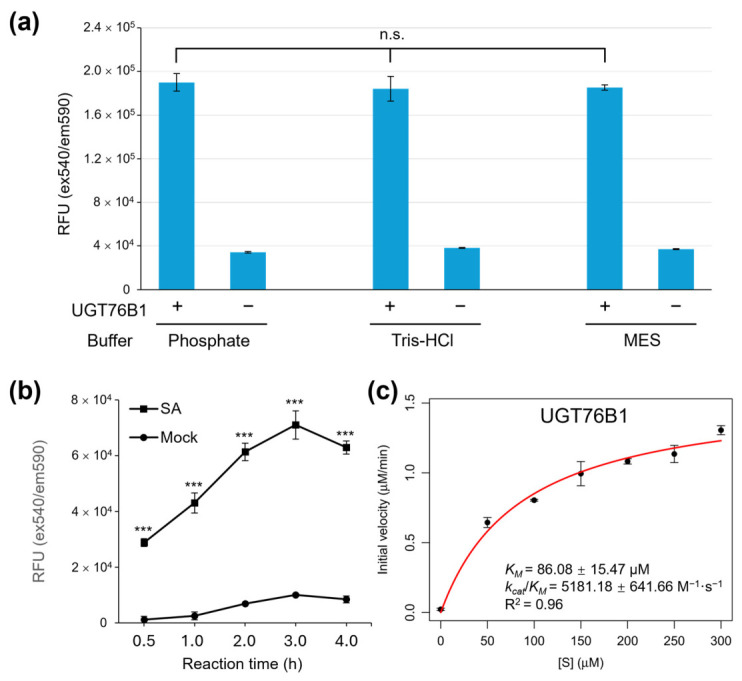
Validation and optimization of the enzyme-coupled fluorescence assay for in vitro glycosyltransferase activity. (**a**) The catalytic activity of UGT76B1 towards salicylic acid (SA) was similarly detected by any of the three buffers (phosphate, Tris-HCl, and MES). Relative fluorescence units (RFUs) were measured at excitation/emission wavelengths of 540/590 nm. The plus sign (+) indicates the presence of the recombinant UGT76B1 enzyme, whereas the minus sign (−) represents the no-enzyme control. Bars represent mean ± SEM (*n* = 3). n.s. indicates no significant difference among the indicated groups (one-way ANOVA followed by Tukey’s HSD post hoc test, *p* > 0.05). (**b**) Time-course analysis of UGT76B1 glycosylation activity in phosphate buffer. The horizontal axis represents the duration of the primary glycosylation reaction, which was terminated by heat inactivation at 95 °C for 5 min. For all indicated time points, the subsequent two-step fluorescence quantitative detection (Step 1 and Step 2, as detailed in Figure S1) was performed under identical standard conditions. The fluorescence intensity peaked at 3 h. Dots represent mean ± SEM (*n* = 3). Asterisks indicate statistically significant differences between the SA group and the mock control at each corresponding time point (two-way ANOVA followed by Sidak’s multiple comparisons test, ***, *p* < 0.0001). (**c**) Steady-state kinetic analysis of UGT76B1 toward SA. Initial reaction velocities were plotted against varying substrate concentrations ([S]). The solid red line represents the nonlinear regression fit to the Michaelis–Menten equation, which was utilized to calculate the kinetic parameters indicated inside the graph.

The enzyme-coupled fluorescence assay developed by Kumagai et al. enables the sensitive quantification of glycosyltransferase activity (Figure S1) [34]. This assay system quantitatively monitors UDP released from UDP-Glc during UGT-mediated glycosylation reactions with a substrate. UDP is stoichiometrically converted to resorufin through two enzyme-coupled reaction steps, and the resulting resorufin can be quantified by fluorescence measurement. To validate the assay, we first assessed the catalytic activity of UGT76B1 toward SA in three commonly used buffers: phosphate, Tris-HCl, and MES (Figure 1a). We did not detect significant differences in fluorescence intensity among the three buffers tested. Time-course analysis revealed that fluorescence intensity increased as glycosylation reaction time was extended (Figure 1b), reaching a maximum at 3 h. However, the background signal also increased accordingly. Therefore, subsequent assays were performed using a reaction time of 2 h and phosphate buffer to minimize the risk of high background fluorescence. Initial reaction velocities measured at varying substrate concentrations were fitted to the Michaelis–Menten equation, yielding a Michaelis constant *K_M_* (SA) = 86.08 ± 15.47 μM and a catalytic efficiency of *k_cat_*/*K_M_* (SA) = 5181.18 ± 641.66 M^−1^·s^−1^. For accurate monitoring of enzyme reactions using this assay system, it is important to determine whether, and to what extent, each substrate affects the two-step reaction that equivalently converts UDP to resorufin. To address this, UDP-to-resorufin conversion was monitored in the presence of each acceptor substrate (SA, NHP, Pip, 3,4-DHBA, or hydroquinone) at 200 μM, which corresponds to the concentration used in the reaction mixture (Figure S2a–e). In the presence of SA, Pip, and 3,4-DHBA, no inhibition of the enzyme-coupled reaction was observed (Figure S2a,c,d). Although SA, 3,4-DHBA, and hydroquinone exhibit autofluorescence, their reported emission wavelengths did not overlap substantially with the resorufin detection wavelength of 590 nm used in this study. Because NHP and Pip lack aromatic chromophores, they are not expected to showed little or no interference with the enzyme-coupled conversion of UDP to resorufin under the conditions relevant to the activity assay. However, in the UDP standard curve generated in the presence of NHP, a slight but significant deviation was detected when 100 μM UDP was added (Figure S2b). Because the reaction mixture contained UDP-glucose at a concentration of 100 μM, and UDP-glucose would not be fully converted to UDP during the reaction, this effect was considered negligible even when if NHP glycosyltransferase activity was present. In contrast, the addition of hydroquinone significantly decreased the fluorescence intensity compared with the mock baseline (Figure S2e). This decrease may be attributable to assay interference or fluorescence quenching arising from the chemical properties of hydroquinone. Even under these conditions, the Michaelis constant could be determined using a corrected calibration curve (*K_M_* (hydroquinone) = 55.92 ± 9.57 μM; Figure S3). The initial velocity decreased at higher hydroquinone concentrations even after correction. This non-canonical kinetic profile may reflect substrate inhibition and/or residual interference of hydroquinone with the enzyme-coupled assay system. Therefore, the kinetic parameter estimated for hydroquinone should be interpreted as an apparent *K_M_* value under the tested assay conditions. These results indicate that the enzyme-coupled fluorescence assay used in this study is suitable for detecting the in vitro glycosylation activity of UGTs under the tested conditions.

**Figure 2:**
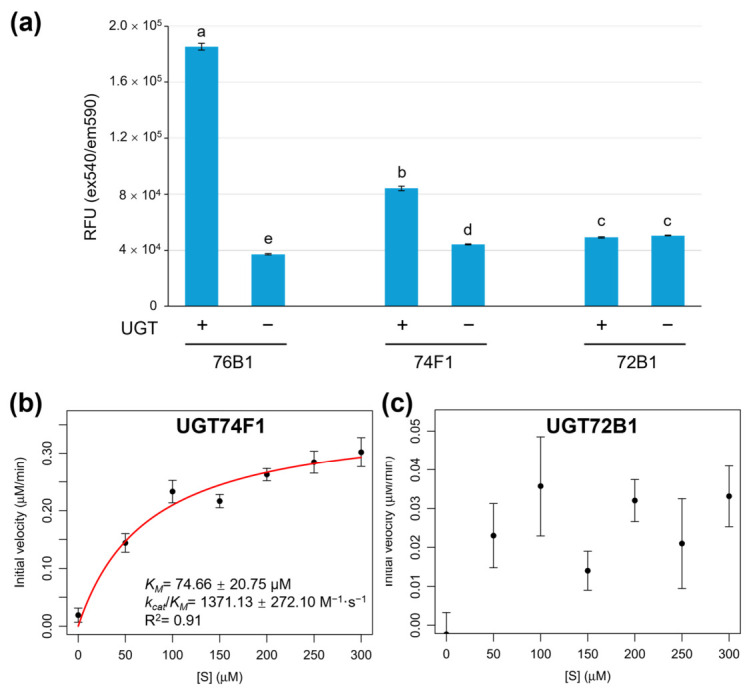
Evaluation of in vitro SA glycosylation activity and steady-state kinetic analysis of previously reported UGTs. (**a**) Relative glycosylation activity of recombinant UGT76B1, UGT74F1 and UGT72B1 toward SA. The catalytic activity of UGT72B1 towards SA was below the detection threshold. RFUs were measured at excitation/emission wavelengths of 540/590 nm. The plus sign (+) indicates the presence of the respective recombinant enzyme, whereas the minus sign (−) represents the no-enzyme control. Bars represent mean ± SEM (*n* = 3). Different lowercase letters above the bars indicate statistically significant differences (one-way ANOVA followed by Tukey’s HSD post hoc test, *p* < 0.05). Steady-state kinetic analysis of UGT74F1 (**b**) and UGT72B1 (**c**) toward SA. Initial reaction velocities were plotted against varying substrate concentrations ([S]). The solid red line represents the nonlinear regression fit to the Michaelis–Menten equation. While UGT74F1 exhibited typical saturation kinetics, allowing for the determination of kinetic parameters (*K_M_* and *k_cat_*/*K_M_*), UGT72B1 displayed marginal activity and poor curve fitting where no biologically meaningful Michaelis constant (*K_M_*) or specificity constant (*k_cat_*/*K_M_*) could be determined.

Next, UGT74F1 and UGT72B1 were included as positive and negative controls to further validate the assay. UGT74F1 exhibited detectable in vitro glycosylation activity toward SA, but both *K_M_* and *k_cat_*/*K_M_* were lower than those of UGT76B1 (Figure 2a,b). Conversely, the fluorescence signal of UGT72B1 in the presence of SA did not significantly deviate from the no-enzyme mock control, and the failure to fit the resulting data to the Michaelis–Menten model further confirmed its lack of SA glycosylation activity (Figure 2a,c).

### 3.2. UGT76B1 Exhibits Negligible Glycosyltransferase Activity Toward NHP, Compared to SA

**Figure 3:**
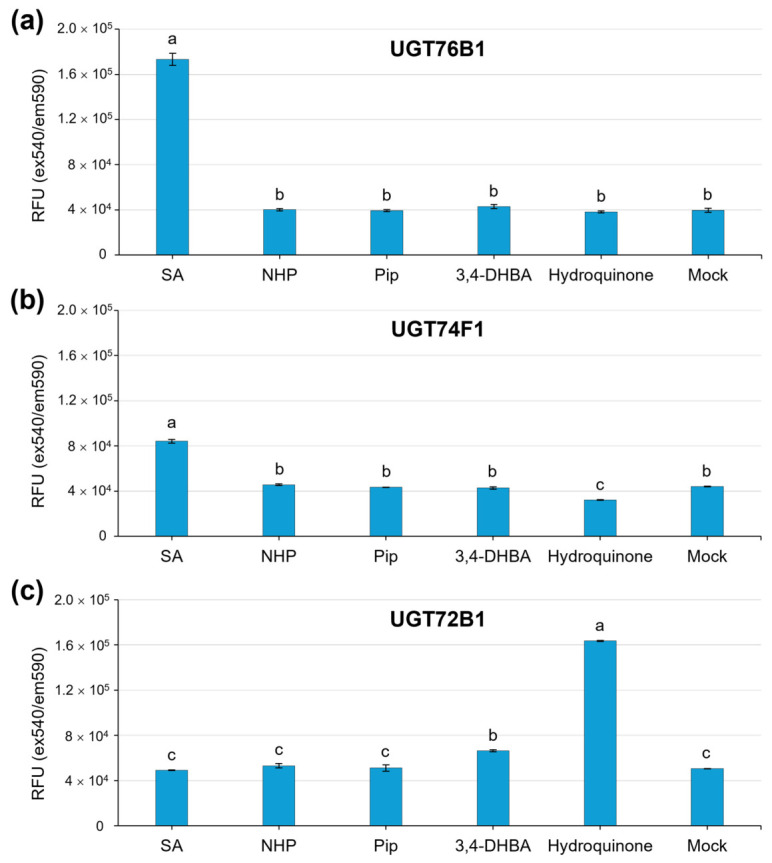
Substrate specificity profiling of recombinant UGT76B1, UGT74F1, and UGT72B1. (**a**–**c**) In vitro glycosylation activities of UGT76B1 (**a**), UGT74F1 (**b**), and UGT72B1 (**c**) were evaluated against a panel of candidate substrates: SA, *N*-hydroxypipecolic acid (NHP), pipecolic acid (Pip), 3,4-dihydroxybenzoic acid (3,4-DHBA), and hydroquinone. Mock reactions using H_2_O as negative controls. None of the three UGTs exhibited catalytic activity toward NHP. RFUs were measured at excitation/emission wavelengths of 540/590 nm. Bars represent mean ± SEM (*n* = 3). Different lowercase letters above the bars indicate statistically significant differences among the diverse substrate treatments (one-way ANOVA followed by Tukey’s HSD post hoc test, *p* < 0.05).

To determine the substrate specificity of these three UGTs—UGT76B1, UGT74F1, and UGT72B1—we employed the same fluorescence-based assay to measure fluorescence intensity in the presence of SA, NHP, Pip, 3,4-dihydroxybenzoic acid (3,4-DHBA), and hydroquinone. Surprisingly, and in stark contrast to previous reports, UGT76B1 did not exhibit glycosylation activity toward NHP (Figure 3a). UGT74F1 displayed glycosylation activity only toward SA (Figure 3b), whereas UGT72B1 catalyzed the glycosylation of 3,4-DHBA and exhibited strong catalytic activity toward hydroquinone. These results are consistent with previous reports [20,27,28,35].

Since we could not detect the expected activity of UGT76B1 toward NHP in our in vitro assay system, we questioned the biological activity of the NHP used in this study. In this study, we utilized commercially available NHP, which is distinct from the chemical synthesized NHP employed in several previous studies [5,29,30,31,32]. To further validate this result, we compared NHP compounds obtained from three different chemical vendors—T19458 from TargetMol (Boston, MA, USA), ATEH9888495A from AstaTech Inc. (Bristol, PA, USA), and HY-N7378 from MedChemExpress (Monmouth Junction, NJ, USA)—and obtained consistent results across all samples (Figure S4a). Although it remains possible that all three preparations, likely produced using similar synthetic approaches, were biologically inactive, we therefore tested the ability to induce defense gene expression in plants. When NHP was infiltrated into rosette leaves, the marker gene *PR1* was significantly induced in the treated leaves at 24 hpt, confirming the biological activity of the commercially available NHP (Figure S4b). SA treatment also elicited a similar response in rosette leaves (Figure S4c). Under these experimental conditions, *PR1* was also weakly induced in distal, untreated leaves at 24 hpt following NHP treatment (Figure S4b). These results indicate that the commercial NHP used in this study contained a biologically active form and/or component capable of inducing defense gene expression in vivo. However, this bioassay does not verify its stereochemical purity or its suitability as a substrate for enzymatic reactions in vitro.

### 3.3. Evaluation of NHP Glycosylation Activities of Candidate UGTs

**Figure 4:**
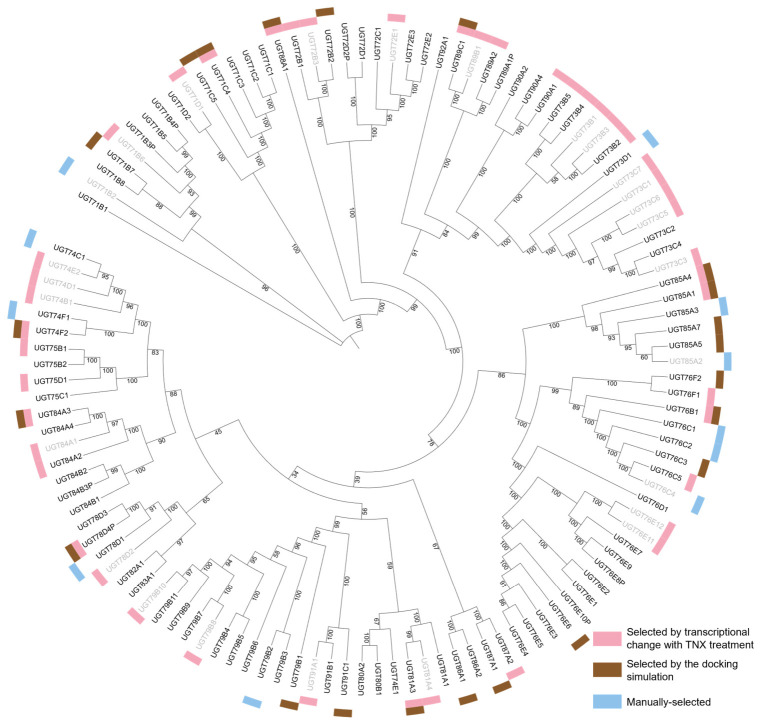
Candidate selection of Arabidopsis UGTs for NHP glycosylation. The phylogenetic tree of 120 UGTs in *A. thaliana* was constructed utilizing maximum-likelihood estimation via IQ-TREE and visualized using iTOL. Candidates identified through distinct strategies are mapped onto the tree with color-coded annotations: pink indicates UGTs whose transcriptional levels are altered by tenoxicam (TNX) treatment; brown indicates UGTs showing a certain level of binding affinity for NHP in the molecular docking simulations; and blue indicates manually selected UGTs chosen based on their phylogenetic proximity to UGT76B1, their positions within phylogenetic gaps, or previous reports. Numbers at the branch nodes represent bootstrap support values. Additionally, UGTs shown in gray font represent candidates that could not be successfully expressed as recombinant proteins in this study.

The negligible activity of UGT76B1 toward NHP is insufficient to account for the physiological conversion of NHP, implying that alternative NHP glycosyltransferases may exist within the Arabidopsis UGT family. Previous transcriptome analyses have revealed that TNX broadly represses SA-inducible, NPR1-dependent gene expression [33]. Given that UGT expression is dynamically modulated by biotic and abiotic stresses to regulate plant immunity [25,30,32,33,36], we interrogated the transcriptomic landscape of TNX-treated plants to identify potential NHP glycosyltransferase candidates. A total of 37 UGTs, including UGT76B1, were identified as significantly upregulated following TNX treatment (Figure 4 and Table S3), corroborating its established function in SA-induced immunity [27,28,33,36]. Conversely, eight UGTs were observed to be significantly downregulated (Table S3). To ensure a comprehensive screening pool, these repressed candidates were also selected for further functional investigation alongside the upregulated group.

Complementary to the transcriptomic approach, an in silico structure-based screening was conducted to identify additional candidate UGTs with potential activity toward NHP. The three-dimensional structures of all UGTs in *A. thaliana* were analyzed using AlphaFold3, and their binding affinities for NHP were assessed using AutoDock Vina [37]. Based on the simulation results, 21 UGTs exhibiting the most favorable binding energies were prioritized for experimental testing (Table S3). Although the extent to which this type of simulation reflects the actual situation remains unclear, we used these UGTs as candidates for further verification of their in vitro enzymatic activities. Note that UGT71D2 was not included because it did not meet the criteria in the initial calculation but met them after recalculation. All candidates were mapped onto a comprehensive UGT phylogenetic tree (Figure 4). To fill the phylogenetic gaps, ten additional UGT genes were manually selected, particularly in proximity to UGT76B1, in addition to previously analyzed enzymes such as UGT74F1.

**Figure 5:**
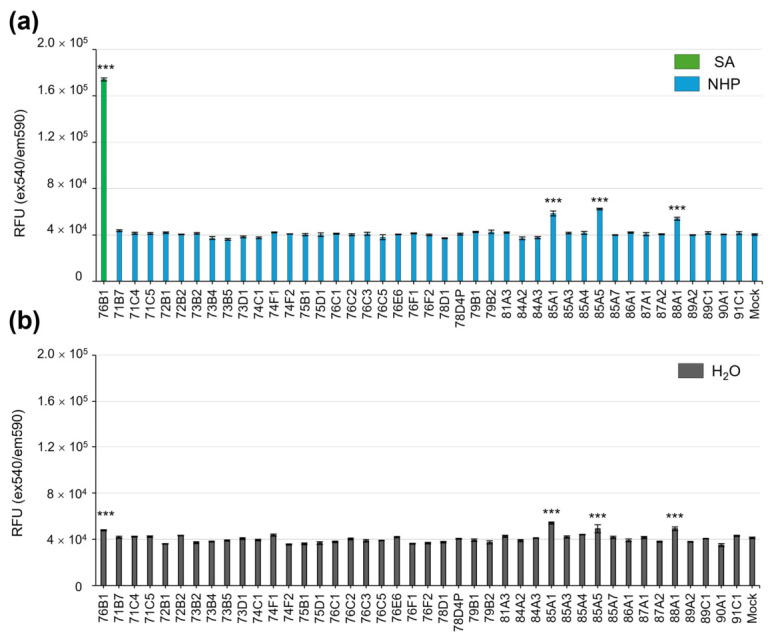
Glycosylation activity of selected candidate UGTs toward NHP. (**a**) Enzymatic activities measured using the in vitro assay system toward NHP are shown as blue bars. The robust activity of UGT76B1 towards salicylic acid (SA), shown as a green bar, is included as a positive control. (**b**) Corresponding substrate-independent background activity of each UGT, evaluated by substituting the substrate with H_2_O. Relative fluorescence units (RFUs) were measured at excitation/emission wavelengths of 540/590 nm. Bars represent the mean ± SEM. Asterisks indicate statistically significant differences compared to the no-enzyme mock control (one-way ANOVA followed by Dunnett’s post hoc test; ***, *p* < 0.001). Although UGT85A1, UGT85A5, and UGT88A1 exhibited statistically significant signal increases in the presence of NHP, these were attributable to substrate-independent background noise rather than authentic NHP glycosylation.

Among the 68 selected candidate genes, excluding duplicates, a total of 46 UGTs, including UGT76B1, were initially expressed and purified using a heterologous expression system in *Escherichia coli* ArcticExpress (DE3). However, the target bands for 4 UGTs, 71B2, 73B3, 76C4, and 85A2, were not detected by SDS-PAGE; therefore, 42 UGTs genes were considered successfully expressed/purified for further analysis (Figure S6). Protein expression of the remaining 22 genes has not yet been tested. The purified recombinant UGTs were subsequently subjected to the established enzyme-coupled fluorescence assay to evaluate their potential glycosylation activity toward NHP. Among all tested candidate enzymes, only UGT85A1, UGT85A5, and UGT88A1 showed statistically significant increases in fluorescence intensity relative to the mock control when NHP was provided as a substrate (Figure 5a). However, proportional increases in background fluorescence signals were also detected in the substrate-free control reactions (Figure 5b), and ultimately no significant differences were observed between these UGTs and their respective controls.

To verify the possibility of trace catalytic function, we attempted steady-state kinetic profiling for these three UGTs as well as UGT76B1. Consistent with our assumption, the resulting reaction velocity data exhibited high variance and completely failed to conform to the Michaelis–Menten equation, rendering the extraction of valid kinetic parameters mathematically impossible (Figure S5). In conclusion, these comprehensive in vitro analyses demonstrate that none of the selected candidate UGTs possess functional NHP glycosylation activity under standard assay conditions.

### 3.4. Effects of Alternative Sugar Donors and Divalent Cation Cofactors on Glycosylation Activity of UGTs Toward NHP

**Figure 6:**
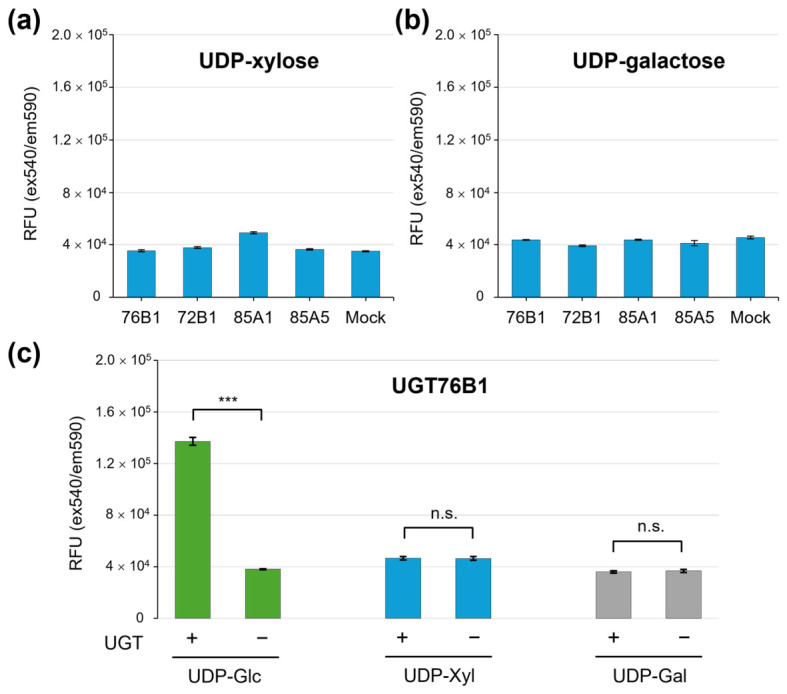
Evaluation of alternative sugar donors on the glycosylation activity of UGTs toward NHP. (**a**,**b**) Glycosylation activity of the four selected UGTs toward NHP was evaluated using UDP-xylose (**a**) or UDP-galactose (**b**) instead of UDP-glucose as the sugar donor. (**c**) Glycosylation activity of UGT76B1 toward SA was evaluated using UDP-glucose, UDP-xylose or UDP-galactose as sugar donors. The plus sign (+) indicates the presence of the recombinant UGT76B1 enzyme, whereas the minus sign (−) represents the corresponding no-enzyme control. RFUs were measured at excitation/emission wavelengths of 540 nm/590 nm after reaction in the in vitro assay system. Bars represent the mean ± SEM. Asterisks indicate statistically significant differences between the enzyme-containing reaction and its corresponding no-enzyme control (one-way ANOVA followed by Tukey’s HSD post hoc test, ***, *p* < 0.001; n.s., not significant).

UGT76B1 belongs to glycosyltransferase family 1 (GT1) and exhibits a typical GT-B-fold structure containing two Rossmann fold-like domains linked by a deep cleft. This structural arrangement determines its ability to recognize common features across multiple substrates in vitro [38,39,40,41]. Sugar donor specificity of UGTs is dictated by the Plant Secondary Product Glycosyltransferase (PSPG) motif located in the C-terminal domain [42,43]. While most plant UGTs utilize UDP-Glc as their primary sugar donor, the spectrum of nucleotide sugars available in nature is diverse [44,45,46]. Certain members of the UGT72 and UGT84 families are known to accept alternative donors such as UDP-xylose (UDP-Xyl) and UDP-galactose (UDP-Gal) [47]. To investigate whether the observed lack of NHP glycosylation was due to a sugar donor mismatch, we evaluated UDP-Xyl and UDP-Gal as alternative donors in our assay. The in vitro results demonstrated that replacing UDP-Glc with either UDP-Xyl or UDP-Gal did not restore detectable NHP glycosylation activity in the selected UGTs (Figure 6a,b). Furthermore, UGT76B1 could not catalyze SA glycosylation when supplied with these alternative donors, underscoring its strict catalytic requirement for UDP-Glc (Figure 6c). However, this analysis was limited to UDP-Xyl and UDP-Gal. Other biologically relevant UDP-sugar donors, such as UDP-glucuronic acid and UDP-rhamnose, were not examined in this study; therefore, the possibility that UGT76B1 or other UGTs utilize these donors for NHP glycosylation cannot be excluded.

**Figure 7:**
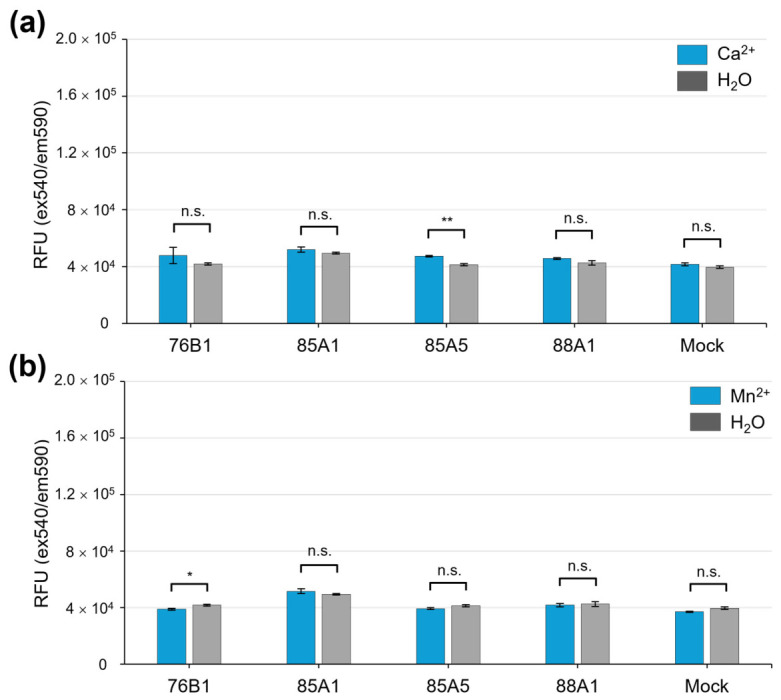
Evaluation of the effects of divalent metal cations on glycosylation activity of UGTs toward NHP. (**a**,**b**) The glycosylation activity of the four selected UGTs toward NHP was evaluated in the presence of Ca^2+^ (1 mM) (**a**) or Mn^2+^ (500 μM) (**b**) instead of Mg^2+^ (10 mM), as indicated by the blue bars. The corresponding background activity, determined by adding H_2_O as a divalent metal-free control, is shown as gray bars. RFU values were measured at excitation/emission wavelengths of 540/590 nm. Bars represent the mean ± SEM. Asterisks indicate statistically significant differences compared with the divalent metal-free control (independent-samples *t*-test, *, *p* < 0.05; **, *p* < 0.01; n.s., not significant). The concentrations of the divalent metals differed because excessive Ca^2+^ or Mn^2+^ caused precipitation in the reaction system. Although the addition of Ca^2+^ to UGT85A5 and Mn^2+^ to UGT76B1 produced slight increases in RFUs compared with the metal-free control, the signal levels were too low to be considered sufficient evidence of enzymatic activity or to allow reliable calculation of *K_M_* values.

Next, we explored the potential influence of divalent cation cofactors. Although GT-B fold glycosyltransferases are generally less dependent on divalent cations than GT-A fold enzymes, some GT1 family enzymes are influenced by divalent cations [48,49,50,51]. We attempted to replace Mg^2+^ in the reaction buffer for the selected UGTs with Mn^2+^ or Ca^2+^. Supplementation with these divalent cations did not increase RFU in most of the UGTs tested; however, Ca^2+^ addition to UGT85A5 and Mn^2+^ addition to UGT76B1 resulted in slight increases in RFUs compared with the metal-free control (Figure 7a,b). Nevertheless, the signal levels were too low to constitute sufficient evidence of enzymatic activity or to allow reliable calculation of *K_M_* values. Consequently, we concluded that Ca^2+^ and Mn^2+^ did not enhance the detectable glycosylation activity of these UGTs toward NHP under the conditions tested.

## 4. Discussion

In this study, we systematically re-evaluated the enzymatic activities of known and non-reported UGTs potentially involved in the metabolism of NHP, a critical mobile signal associated with plant SAR. However, in contrast to previous reports, we could not detect glycosylation activity of UGT76B1 toward NHP.

One possible reason for this discrepancy is the difference in detection methods. In our experiments, we did not directly measure NHPG in the reaction solution. Instead, we monitored the production of UDP which is generated in equimolar amounts with glycosylated substrates such as NHPG. The *K_M_* value of UGT76B1 toward SA determined using our enzyme-coupled fluorescence assay was 86.08 ± 15.47 μM, which is consistent with that reported by Mohnike et al. [30] and approximately two-fold lower than values reported in other studies [28,52]. This suggests that our method is comparable to other methods and is sufficiently capable of measuring the enzymatic activity of UGTs. Note that the specificity constant of UGT76B1 towards SA determined in our assay (*k_cat_*/*K_M_* = 5181.18 ± 641.66 M^−1^∙s^−1^) was 2.4-, 17-, and 35-fold higher than the values previously reported by Maksym et al. [52], Mohnike et al. [30], and Noutoshi et al. [28], respectively. These differences likely arise from variations in the sensitivity of the respective detection methodologies. The reliability of our enzyme-coupled fluorescence assay has now been validated; therefore, this assay could serve as a useful technique for accurately measuring UGT enzymatic activities, substrate specificity, and kinetic parameters, even when appropriate reference compounds for glycosylated substrates are unavailable for quantitative analyses using methods such as mass spectrometry.

Our assay detected weak UDP production by several Arabidopsis UGTs, including UGT76B1, UGT85A1, UGT85A5, and UGT88A1, in the presence of UDP-Glc alone, even in the absence of the target substrate. It has been reported that some UGTs can hydrolyze UDP-Glc to UDP and glucose using H_2_O instead of an acceptor substrate [53]. The detection of spontaneous UDP-Glc hydrolysis likely reflects the high sensitivity of this assay and may represent substrate-independent background noise. We initially misinterpreted these signals as evidence of NHP glycosylation and therefore spent considerable time validating them. Thus, control experiments without the target substrate are necessary when using this assay.

The lack of significant in vitro activity of UGT76B1 toward NHP observed in our study contrasts with several existing reports claiming that UGT76B1 is a glycosylation enzyme for NHP [29,30,31,32]. All these previous studies relied on mass spectrometry technology for the detection of NHP and NHPG. Because no chemically synthesized authentic standard is currently available for NHPG, the methodologies used for product identification in previous studies have varied and have inherent limitations. Two studies relied on exact mass measurement and fragmentation patterns as indicators for NHPG detection [31,32]. Other studies prepared NHPG enzymatically using NHP and recombinant UGT76B1 as a reference material; however, NHPG concentrations were estimated indirectly [29,30]. All experiments relied on m/z and fragment pattern but no confirmation with NMR. In a situation where uncertainties remain, we evaluated this hypothesis using a different approach that is independent of mass spectrometry technique. In comparison with the obvious glycosylation reaction toward SA, activity of UGT76B1 recombinant protein toward NHP was not detected at all, although weak substrate-independent background level-production of UDP was detected. Our experiments also did not directly measure NHPG, but NHPG should not be generated without producing UDP in the reaction solution. These results indicate that recombinant UGT76B1 alone does not exhibit detectable NHP glycosylation activity under our in vitro conditions tested. One possibility causing this discrepancy could be attributable to the differences in the source of the NHP chemical reference used. NHP is a chiral molecule and stereochemical configuration of the product can affect bioactivity. Also, stability of NHP could affect the result. The NHP chemical product used in our study at least could induce *PR1* gene expression in planta; therefore, it can be expected to contain at least biologically active molecule. Another possible explanation is that UGT76B1 may require plant-specific post-translational modification or interaction with cellular factors for detectable catalytic activity toward NHP. Because the recombinant proteins used in this study were expressed in *E. coli*, such modifications or interactions may not have been fully reproduced in our in vitro assay system. Furthermore, it is important to consider the potential influence of the fusion tag on catalytic activity. All recombinant UGTs evaluated in this study retained an N-terminal 6 × His-tag. Because the N-terminal region of plant UGTs contributes to substrate recognition, the tag may have affected local protein conformational and/or substrate access to the active site. Nevertheless, the same recombinant UGT76B1 showed robust activity toward SA, indicating that the protein was catalytically competent under our assay conditions.

SA has a largely planar structure, in which the benzene ring and the carboxyl group are positioned almost in the same plane. In contrast, NHP contains a saturated piperidine ring that adopts a chair-like conformation, with the carboxyl group projecting three-dimensionally from the ring scaffold. It is therefore reasonable to assume that SA and NHP adopt distinct orientations and interaction modes within the substrate-binding pocket of UGT76B1. Although plant UGTs often exhibit broad substrate tolerance, our enzymatic assays revealed clear substrate preferences even among structurally related small molecules. Thus, structural similarity inferred from two-dimensional representations should not be taken as sufficient evidence that SA and NHP are glycosylated by the same enzyme. Previous observations that endogenous NHP levels are altered in *ugt76b1* mutants, in addition to changes in SA metabolism, may have further supported the proposed involvement of UGT76B1 in NHP glycosylation. However, our biochemical analysis revealed that UGT76B1 also lacks glycosylation activity toward both NHP and the upstream precursor Pip. Therefore, such changes can also be explained by reciprocal activation or coordinated regulation of SA- and NHP-dependent immune metabolic pathways, rather than by direct glycosylation of NHP by UGT76B1.

According to the UGT Nomenclature Committee, 123 UGTs have been identified in the *A. thaliana* genome. However, three UGTs were excluded from this study because they do not encode valid predicted amino acid sequences. We examined the potential NHP glycosylation activity of 42 UGT proteins, representing 35% of the 120 UGT family members in *A. thaliana*. However, none of the tested UGTs showed detectable glycosylation activity toward NHP under our assay conditions. NHP-hexose conjugates, most likely NHPG, has been detected in *A. thaliana* by mass spectrometry [5,6,29,30,31,32], indicating that one or more genes encoding enzyme(s) responsible for the production of these metabolites should exist in the genome. UGT74F1, UGT74F2, and UGT76B1 have been reported to exhibit SA glycosyltransferase activity [20,28,35], although they are positioned in distinct regions of the UGT phylogenetic tree. This suggests that substrate preference is difficult to infer solely from phylogenetic relationships, at least when trees are constructed using full-length protein sequences. In contrast, UGT76B1 was previously selected as a candidate NHP glycosyltransferase based on its transcriptional upregulation upon biotic stress and/or its co-expression with genes involved in NHP biosynthesis [31,32]. In the present study, we also selected candidate UGTs based in part on transcriptional responses to TNX. These genes were distributed broadly across the phylogenetic tree, suggesting that transcriptional regulation has evolved independently of phylogenetic position within the UGT family. Among the 68 genes selected on the basis of transcriptional responses and docking simulations, 22 could not be examined by in vitro assays because recombinant protein expression in *E. coli* was unsuccessful. Therefore, the absence of detectable NHP glycosylation activity among the UGTs tested here does not exclude the presence of an NHP glycosyltransferase in *A. thaliana*. Importantly, the lack of detectable activity within this selected UGT panel should not be evidence against UGT-mediated NHP glycosylation in general. Further screening will be required using both untested candidates and UGTs outside the current selection criteria.

**Figure 8:**
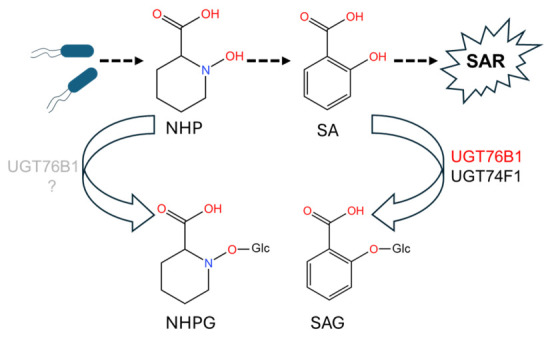
Schematic model of UGT-mediated SA and NHP metabolism during the establishment of SAR. Pathogen infection triggers the accumulation of NHP and SA, which function synergistically. UGT76B1 serves as the primary SA glycosyltransferase (highlighted in red to indicate verified active catalysis) and deactivates SA by converting it into SAG. In contrast, based on our in vitro biochemical analyses, recombinant UGT76B1 lacks direct glycosylation activity toward NHP (indicated by the greyed-out text), suggesting that NHP glycosylation is likely catalyzed by other uncharacterized UGTs, or that UGT76B1 requires post-translational modification or cofactors (represented by the question mark).

Considering the relationship between in vitro catalytic efficiency and in planta physiological relevance, large-scale analyses of enzyme kinetics have demonstrated that the most enzymes do not operate at the theoretical maximum limits, and that many of enzymes with only moderate catalytic efficiencies can be identified in vitro [54]. In plant immune signaling, structural and biochemical studies of tobacco SA-binding protein 2 (SABP2) revealed that while it possesses a relatively low turnover rate for MeSA in vitro, it exhibits high affinity and specificity at physiologically relevant concentrations [55]. Similarly, ENHANCED PSEUDOMONAS SUSCEPTIBILITY 1 (EPS1) exhibits atypical catalytic activity in vitro, yet it is essential for driving the massive accumulation of SA during pathogen infection [56]. Therefore, a weak inherent catalytic activity of a UGT could theoretically be physiologically meaningful in vivo. However, NHP functions as a critical mobile signal, requiring both NHP and NHPG to accumulate rapidly and abundantly to orchestrate systemic immunity during the establishment of SAR [5]. In our assay, any potential specific activity of UGT76B1 toward NHP was obscured by the background noise resulting from UDP-Glc hydrolysis. Therefore, given its negligible in vitro activity, UGT76B1 is unlikely to act as the primary NHP glycosyltransferase. This further emphasizes the likely existence of undiscovered NHP glycosyltransferase in *A. thaliana* (Figure 8).

## Data Availability

All data is available in the manuscript.

## Supplementary Materials

The following supporting information can be downloaded at: https://www.mdpi.com/article/10.3390/life16060992/s1. Figure S1: Schematic representation of the in vitro enzyme-coupled fluorescence assay system for glycosyltransferase activity. The assay consists of a primary glycosylation reaction followed by a two-step quantitative fluorescence detection process. In the glycosylation reaction, a UDP-glycosyltransferase (UGT) catalyzes the transfer of a glucose moiety from a UDP-sugar, such as UDP-glucose (UDP-G), to an acceptor substrate, such as N-hydroxypipecolic acid (NHP), resulting in the stoichiometric release of UDP together with the formation of the corresponding glycosylated product. In Step 1 of the detection phase, the released UDP is converted to UTP by nucleoside diphosphate kinase (NDP kinase), with concomitant conversion of ATP to ADP. In Step 2, the generated ADP is used by ADPhexokinase to phosphorylate glucose, yielding glucose-6-phosphate (G6P). G6P is subsequently oxidized by G6P dehydrogenase, coupled to the reduction of NADP+ to NADPH. Finally, diaphorase uses NADPH to reduce non-fluorescent resazurin to highly fluorescent resorufin, which is quantified at excitation and emission wavelengths of 540 and 590 nm, respectively; Figure S2: Effects of substrates on the two-step conversion of UDP to resorufin in the in vitro enzyme-coupled fluorescence assay system. (a–e) Potential substrate interference activity on the assay system was evaluated using different concentrations of UDP (0–100 μM) in the absence of UGTs. Each panel compares the substrate-free control (Mock) with a parallel reaction containing the indicated substrate: SA (a), NHP (b), Pip (c), 3,4-DHBA (d), and hydroquinone (e). (f) Linear regression analysis plotting the calculated fluorescence inhibition rate against hydroquinone concentration. Relative fluorescence units (RFU) were plotted against UDP concentrations. Data are presented as the mean ± SEM. Statistical significance between the mock and substrate-treated groups at each UDP concentration was determined using one-way ANOVA followed by Dunnett‘s post hoc test (*, *p* < 0.05; **, *p* < 0.01; ***, *p* < 0.001; n.s., not significant). Notably, SA, Pip, and 3,4-DHBA showed no interference with the assay, whereas a high concentration of UDP (100 μM) caused a slight but significant deviation exclusively when NHP was used as the substrate in the standard curves. In contrast, hydroquinone significantly suppressed the fluorescence signal across all tested concentrations; Figure S3: Steady-state kinetic analysis of UGT72B1 toward hydroquinone. Initial reaction velocities were plotted against varying substrate concentrations ([S]). The solid red line represents the nonlinear regression fit to the Michaelis–Menten equation, which was used to calculate the kinetic parameters shown in the graph. Because hydroquinone induced a concentration-dependent quenching of the fluorescence signal, the data were mathematically corrected prior to curve fitting, as detailed in the Materials and Methods; Figure S4: Comparison of NHP products from three different chemical vendors in the in vitro assay system and confirmation of *PR1*-inducing bioactivity in *Arabidopsis thaliana*. (a) In vitro glycosylation activity of UGT76B1 toward commercially available NHP products from different vendors was evaluated with salicylic acid (SA) and pipecolic acid (Pip) as control substrates. RFU values were measured at excitation/emission wavelengths of 540/590 nm and compared with those of reactions without enzyme. Bars represent the mean ± SEM (*n* = 3). Different lowercase letters above the bars indicate statistically significant differences among substrate treatments (one-way ANOVA followed by Tukey’s HSD post hoc test, *p* < 0.05). (b,c) Relative transcript levels of PR1 were quantified by quantitative RT-PCR in rosette leaves and upper leaves treated with NHP (b) or SA (c) at 8 h and 24 hpi, respectively. Gene expression was normalized to the internal control UBQ10. Data are presented as the mean ± SE (*n* = 3). Individual data points represent biological replicates. Statistical significance was determined using a twotailed Student’s *t*-test. Asterisks indicate significant differences between time points within the same plant: *, *p* < 0.05; **, *p* < 0.01; ns, not significant; Figure S5: Evaluation of selected Arabidopsis UGT proteins, including UGT76B1, for NHP glycosylation activity. (a–d) Measurement of reaction velocities for UGT76B1 (a), UGT85A1 (b), UGT85A5 (c), and UGT88A1 (d) using NHP as the substrate. Initial reaction velocities were plotted against varying substrate concentrations ([S]). Data are presented as the mean ± SEM (*n* = 3). None of the recombinant UGTs tested exhibited typical saturation kinetics, and the reaction velocity data showed either high variability or poor curve fitting. Consequently, no biologically meaningful Michaelis constant (*K_M_*) or specificity constant (*kcat*/*K_M_*) could be determined. Therefore, these data are provided as supportive negative evidence for the absence of observable kinetic activity toward NHP in the in vitro assay. Therefore, these data are provided as supportive negative evidence for the absence of observable kinetic activity toward NHP in the in vitro assay; Figure S6: SDS-PAGE analysis of recombinant UGT proteins after affinity purification. The red frame indicates the target band, and the numbers above each lane represent the predicted molecule weight of the target proteins. UGTs that were not successfully expressed under our experimental conditions are indicated in red text; Table S1: FASTA files of codon-optimized sequences of UGT gene sequences for recombinant protein expression in *Escherichia coli*; Table S2: Summary of amino acid residues of Arabidopsis UGT proteins used for docking simulations with NHP using AutoDock Vina; Table S3: Summary of Arabidopsis UGT genes tested in this study: TNX responsiveness, NHP docking simulation, and recombinant protein expression in *E. coli*.
