# Supplementary material for: UGT76B1 and 41 Additional Arabidopsis UDP-Glycosyltransferases Show No Detectable In Vitro Glycosylation Activity Toward N-Hydroxypipecolic Acid
Source: Life (Basel). 2026 Jun 12;16(6):992. doi: 10.3390/life16060992 (PMC13300909; doi:10.3390/life16060992)
Supplement: Supplementary file 1 [file life-16-00992-s001.zip › Supplementary Figures_Bae et al_Life_260611.pdf]

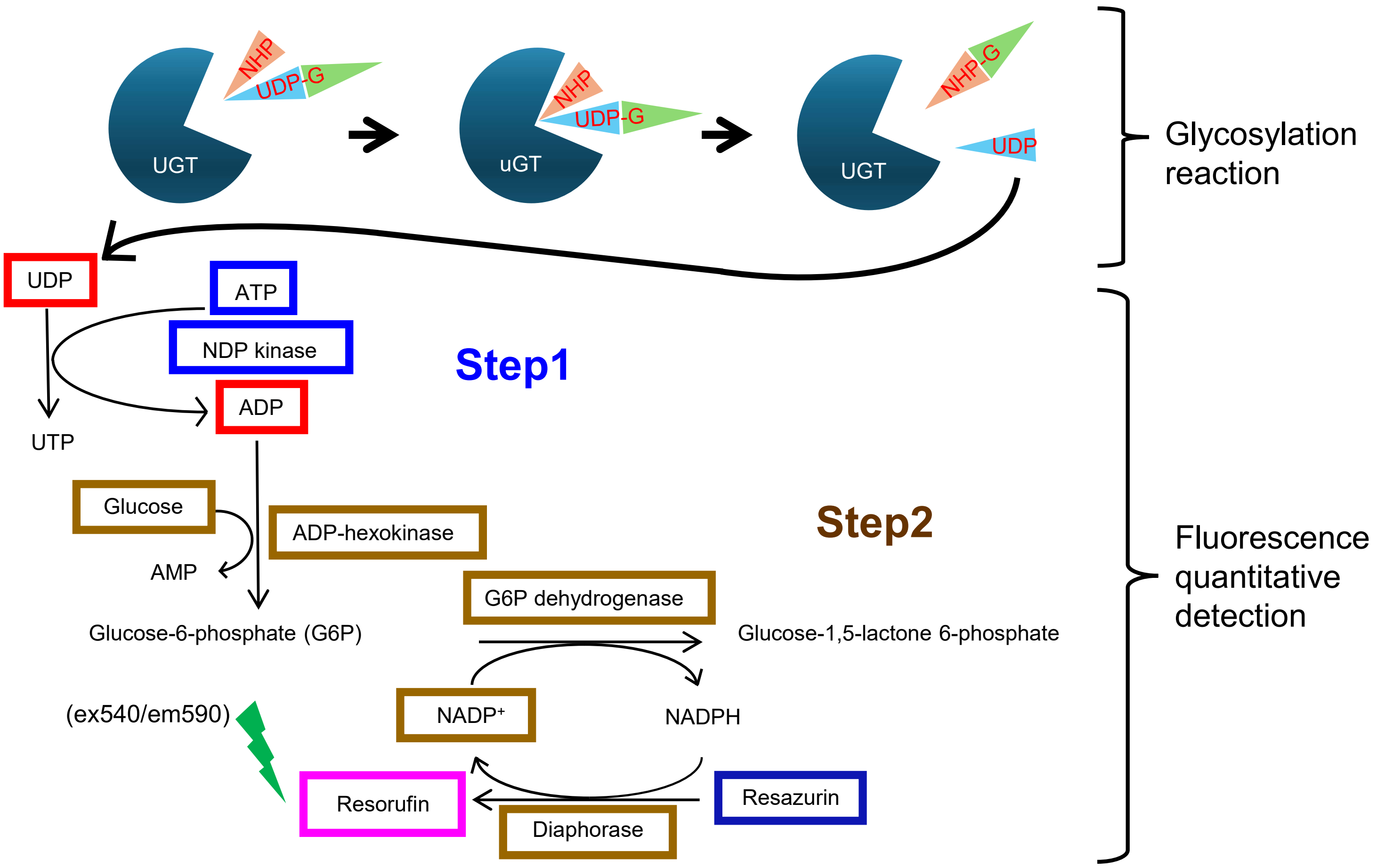

Figure S1. Schematic representation of the in vitro enzyme-coupled fluorescence assay system for glycosyltransferase activity. The assay consists of a primary glycosylation reaction followed by a two-step quantitative fluorescence detection process. In the glycosylation reaction, a UDP-glycosyltransferase (UGT) catalyzes the transfer of a glucose moiety from a UDP-sugar, such as UDP-glucose (UDP-G), to an acceptor substrate, such as *N*-hydroxypipecolic acid (NHP), resulting in the stoichiometric release of UDP together with the formation of the corresponding glycosylated product. In Step 1 of the detection phase, the released UDP is converted to UTP by nucleoside diphosphate kinase (NDP kinase), with concomitant conversion of ATP to ADP. In Step 2, the generated ADP is used by ADP-hexokinase to phosphorylate glucose, yielding glucose-6-phosphate (G6P). G6P is subsequently oxidized by G6P dehydrogenase, coupled to the reduction of $NADP^+$ to NADPH. Finally, diaphorase uses NADPH to reduce non-fluorescent resazurin to highly fluorescent resorufin, which is quantified at excitation and emission wavelengths of 540 and 590 nm, respectively.

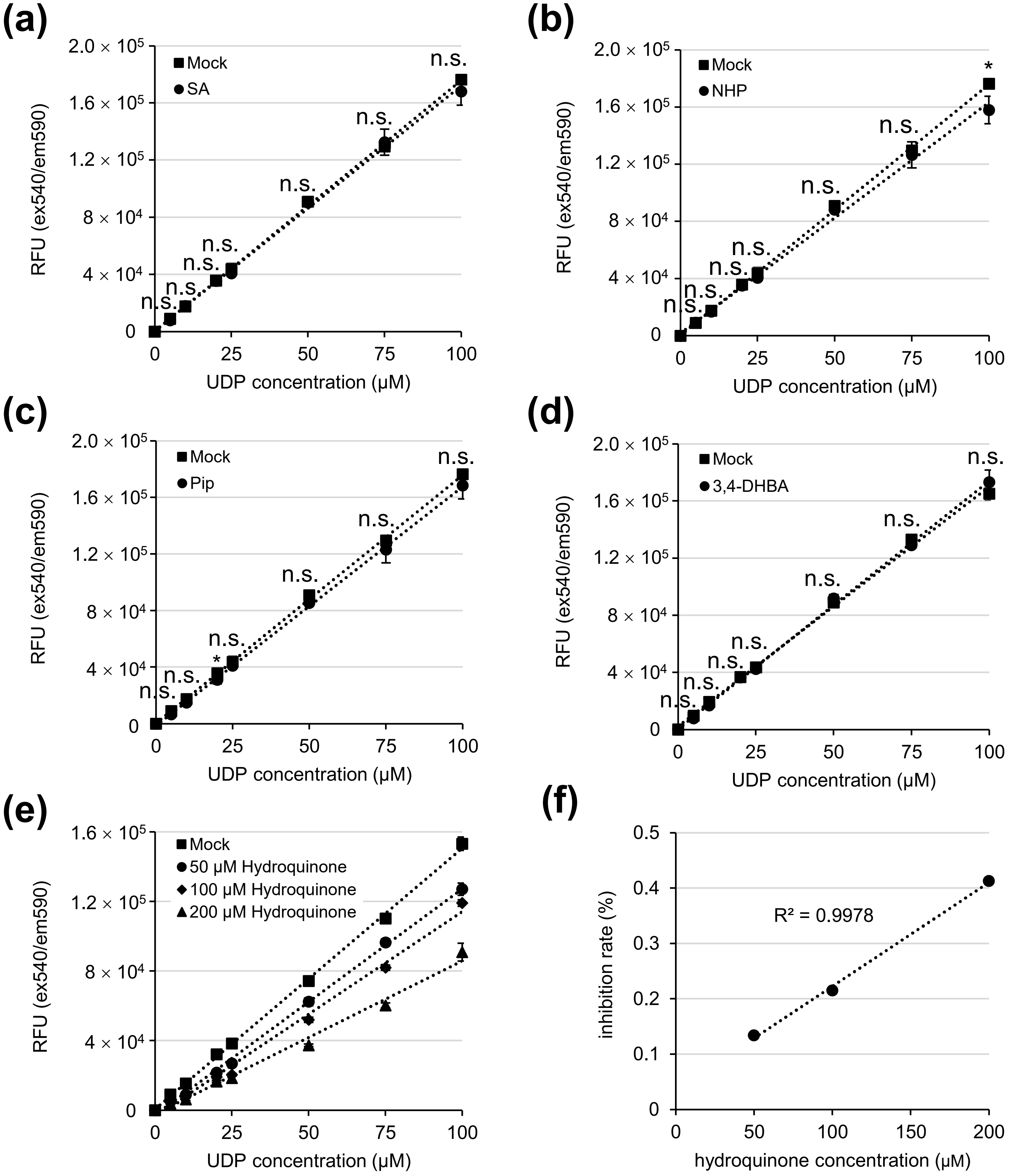

Figure S2. Effects of substrates on the two-step conversion of UDP to resorufin in the in vitro enzyme-coupled fluorescence assay system. (a–e) Potential substrate interference activity on the assay system was evaluated using different concentrations of UDP (0–100 μM) in the absence of UGTs. Each panel compares the substrate-free control (Mock) with a parallel reaction containing the indicated substrate: SA (a), NHP (b), Pip (c), 3,4-DHBA (d), and hydroquinone (e). (f) Linear regression analysis plotting the calculated fluorescence inhibition rate against hydroquinone concentration. Relative fluorescence units (RFU) were plotted against UDP concentrations. Data are presented as the mean ± SEM. Statistical significance between the mock and substrate-treated groups at each UDP concentration was determined using one-way ANOVA followed by Dunnett's post hoc test (*, $P < 0.05$; **, $P < 0.01$; ***, $P < 0.001$; n.s., not significant). Notably, SA, Pip, and 3,4-DHBA showed no interference with the assay, whereas a high concentration of UDP (100 μM) caused a slight but significant deviation exclusively when NHP was used as the substrate in the standard curves. In contrast, hydroquinone significantly suppressed the fluorescence signal across all tested concentrations.

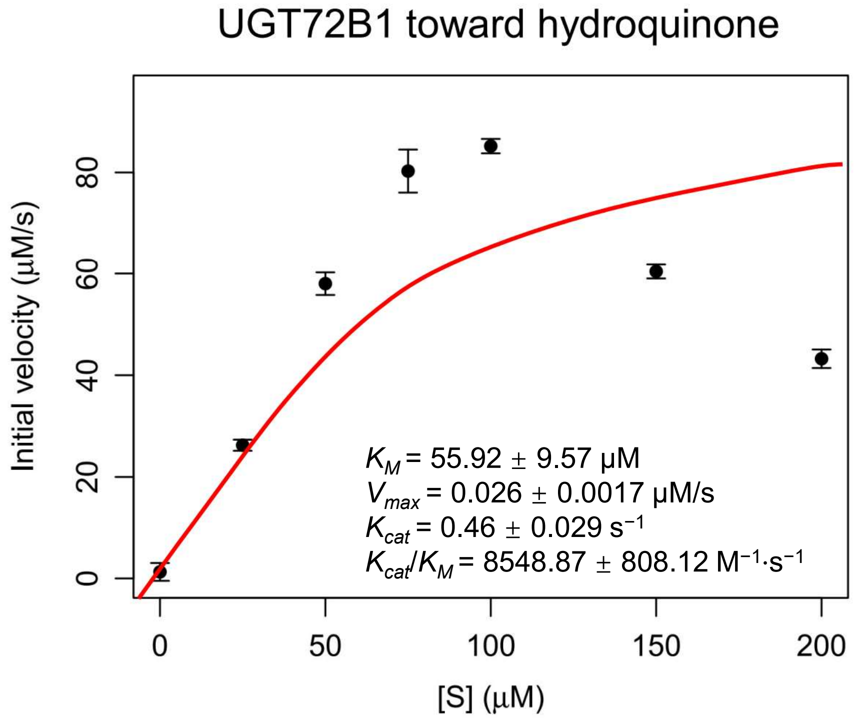

Figure S3. Steady-state kinetic analysis of UGT72B1 toward hydroquinone. Initial reaction velocities were plotted against varying substrate concentrations ([S]). The solid red line represents the nonlinear regression fit to the Michaelis–Menten equation, which was used to calculate the kinetic parameters shown in the graph. Because hydroquinone induced a concentration-dependent quenching of the fluorescence signal, the data were mathematically corrected prior to curve fitting, as detailed in the Materials and Methods.

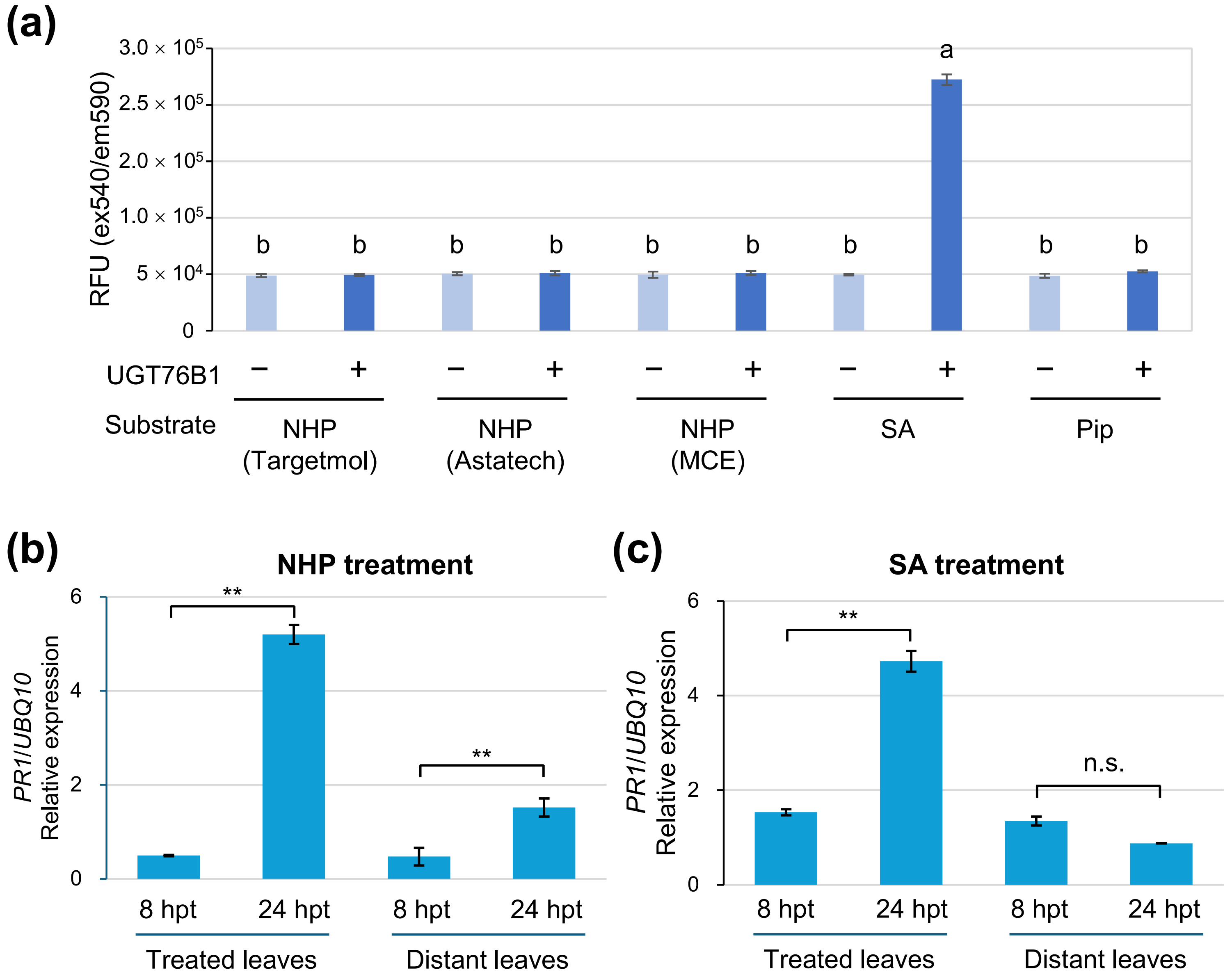

Figure S4. Comparison of NHP products from three different chemical vendors in the in vitro assay system and confirmation of *PR1*-inducing bioactivity in *Arabidopsis thaliana*. (a) In vitro glycosylation activity of UGT76B1 toward commercially available NHP products from different vendors was evaluated with salicylic acid (SA) and pipecolic acid (Pip) as control substrates. RFU values were measured at excitation/emission wavelengths of 540/590 nm and compared with those of reactions without enzyme. Bars represent the mean ± SEM (n = 3). Different lowercase letters above the bars indicate statistically significant differences among substrate treatments (one-way ANOVA followed by Tukey's HSD post hoc test, P < 0.05). (b,c) Relative transcript levels of *PR1* were quantified by quantitative RT-PCR in rosette leaves and upper leaves treated with NHP (b) or SA (c) at 8 h and 24 hpi, respectively. Gene expression was normalized to the internal control *UBQ10*. Data are presented as the mean ± SE (n = 3). Individual data points represent biological replicates. Statistical significance was determined using a two-tailed Student's *t*-test. Asterisks indicate significant differences between time points within the same plant: *, P < 0.05; **, P < 0.01; ns, not significant.

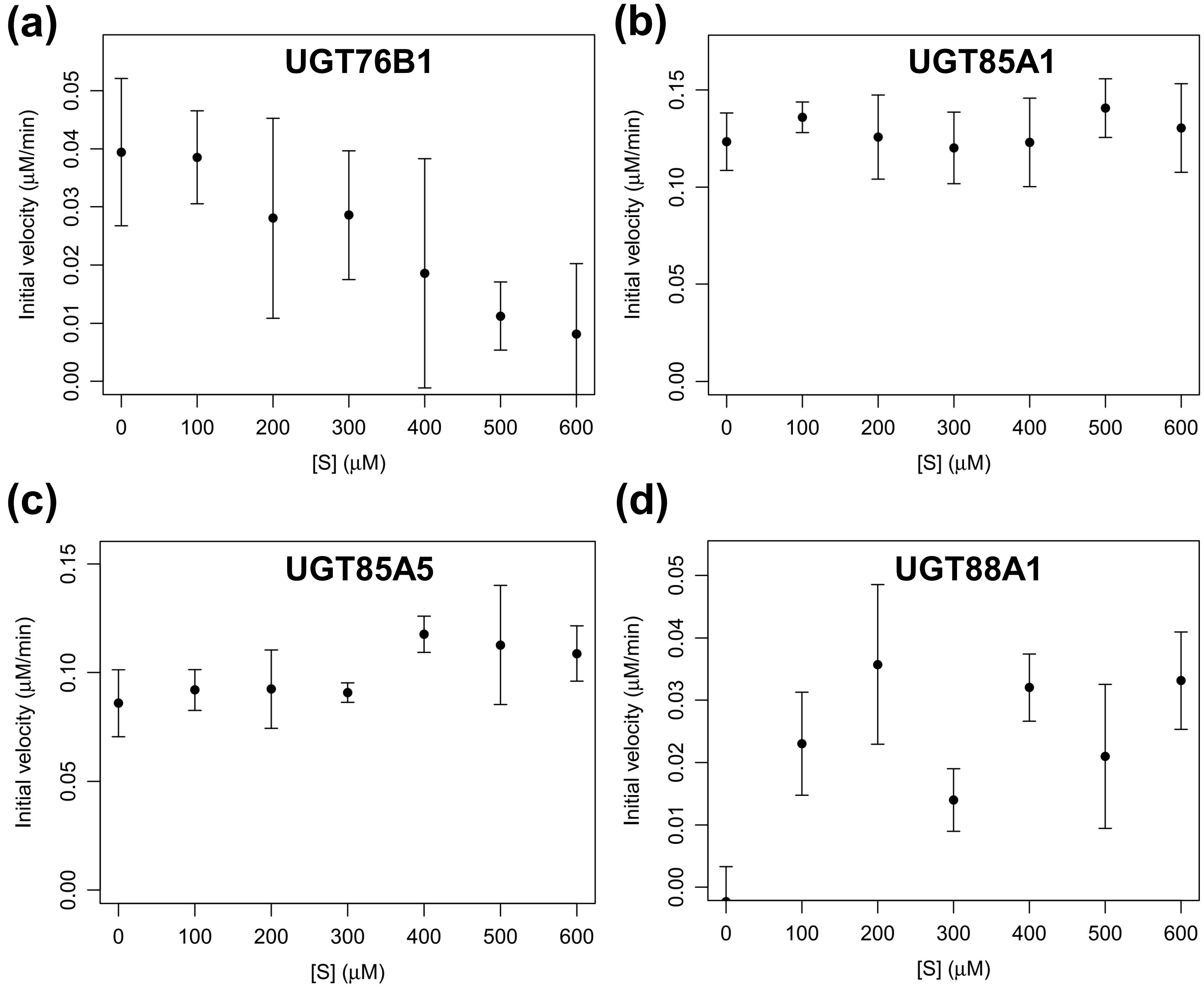

Figure S5. **Evaluation of selected Arabidopsis UGT proteins, including UGT76B1, for NHP glycosylation activity.** (a–d) Measurement of reaction velocities for UGT76B1 (a), UGT85A1 (b), UGT85A5 (c), and UGT88A1 (d) using NHP as the substrate. Initial reaction velocities were plotted against varying substrate concentrations ([S]). Data are presented as the mean ± SEM (*n* = 3). None of the recombinant UGTs tested exhibited typical saturation kinetics, and the reaction velocity data showed either high variability or poor curve fitting. Consequently, no biologically meaningful Michaelis constant (K_M) or specificity constant ($kcat/K_M$) could be determined. Therefore, these data are provided as supportive negative evidence for the absence of observable kinetic activity toward NHP in the in vitro assay. Therefore, these data are provided as supportive negative evidence for the absence of observable kinetic activity toward NHP in the in vitro assay.

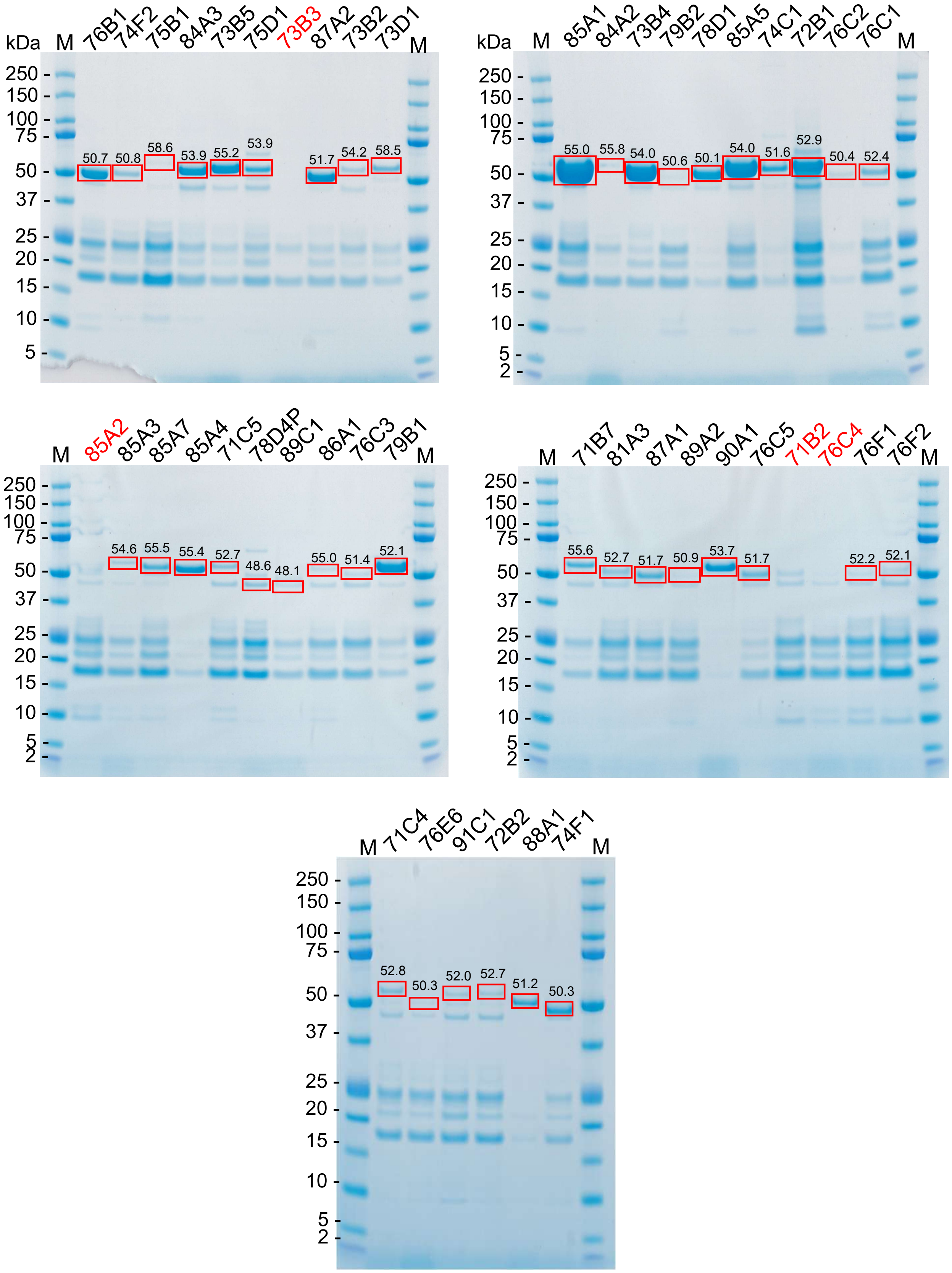

Figure S6. SDS-PAGE analysis of recombinant UGT proteins after affinity purification. The red frame indicates the target band, and the numbers above each lane represent the predicted molecule weight of the target proteins. UGTs that were not successfully expressed under our experimental conditions are indicated in red text.
